# Impact of cerebrovascular stroke on inflammatory periodontal indices: a systematic review with meta-analysis and trial sequential analysis of case-control studies

**DOI:** 10.3389/froh.2024.1473744

**Published:** 2024-10-24

**Authors:** Mario Dioguardi, Maria Eleonora Bizzoca, Stefania Cantore, Giorgia Apollonia Caloro, Gennaro Musella, Filiberto Mastrangelo, Lorenzo Lo Muzio, Andrea Ballini

**Affiliations:** ^1^ Department of Clinical and Experimental Medicine, University of Foggia, Foggia, Italy; ^2^ Department of Precision Medicine, University of Campania “Luigi Vanvitelli”, Naples, Italy; ^3^ Unità Operativa Nefrologia e Dialisi, Presidio Ospedaliero Scorrano, ASL (Azienda Sanitaria Locale) Lecce, Scorrano, Italy

**Keywords:** stroke, periodontitis, brain, oral and dental health, bone loss, oral inflammation, risk factor, tooth loss

## Abstract

**Introduction:**

Cerebrovascular stroke, a leading cause of global morbidity and mortality, is influenced by several modifiable risk factors such as hypertension, diabetes, and smoking. Emerging evidence highlights the significant role of inflammation in stroke pathogenesis, with conditions like periodontitis potentially exacerbating this risk. The aim of this systematic review was to identify and quantify the real impact of periodontal disease in individuals with cerebral stroke through the analysis of inflammatory periodontal indices

**Methods:**

Following PRISMA guidelines, we conducted a systematic review and meta-analysis of case-control studies assessing periodontal indices in stroke patients. Data sources included PubMed, Scopus, and Cochrane Library, with searches extended to grey literature. The review protocol was registered on PROSPERO (CRD42024529767). Studies were evaluated using the Newcastle-Ottawa Scale to assess risk of bias, and meta-analyses were conducted using Review Manager 5.4 and TSA software.

**Results:**

The review included seven case-control studies, comprising 723 stroke patients and 787 controls. Meta-analyses revealed significant differences between stroke and control groups in clinical attachment loss [MD 1.04 mm, 95% CI (0.54, 1.54)], probing pocket depth [MD 0.68 mm, 95% CI (0.31, 10.6)], and radiological bone loss (MD 2.15 mm, 95% CI [−1.58, 5.89]. These findings indicate that stroke patients exhibit worse periodontal health compared to controls, supporting a potential link between periodontal inflammation and stroke.

**Conclusion:**

This study confirms the significant impact of periodontal disease on stroke patients and highlights the importance of oral health in preventing adverse cerebrovascular events.

**Systematic Review Registration:**

PROSPERO, identifier (CRD42024529767).

## 1 Introduction

Ischemic stroke is a leading global cause of death and disability, with its prevalence notably rising in developing countries (1). This condition results from a significant reduction in cerebral blood flow, impairing the delivery of oxygen and essential nutrients, leading to substantial brain damage (2, 3). Such damage manifests as ischemic stroke or cerebral ischemia when a vessel is occluded, or as hemorrhagic stroke when a vessel ruptures (4). In both instances, nerve cells in the affected brain region are deprived of oxygen and may die within minutes of hypoxia onset, causing impairments in bodily functions, including motor skills, language, and memory, depending on the specific brain region involved. The primary etiological factors of ischemic stroke include atherosclerosis, thrombosis, embolism, vascular diseases, autoimmune diseases, and trauma (5, 6). Modifiable risk factors encompass hypertension, diabetes, obesity, hypercholesterolemia, sedentary lifestyle, smoking, and alcohol abuse (7). Emerging evidence underscores the pivotal role of inflammation in stroke development, with inflammatory conditions of the oral cavity potentially playing a fundamental role (8). Periodontitis, as defined by the latest World Workshop in 2017, is a chronic multifactorial inflammatory disease associated with dysbiotic plaque biofilms and characterized by the progressive destruction of the dental apparatus (9). According to the recent epidemiological study from 2023, estimated pooled prevalence of periodontitis is nearly 60%, with its severe stage affecting approximately 24% (10). Recent studies have linked periodontitis to various systemic conditions, such as arthritis, type 2 diabetes, Alzheimer's disease, respiratory infections, adverse pregnancy outcomes, and atherosclerosis (11–13). The explanation for these associations has been extensively studied, with experimental models suggesting that periodontitis contributes to systemic inflammation through both direct and indirect routes (14). The direct route involves ulceration the epithelium of the periodontal pockets, which can become a passage for bacteria into the systemic circulation leading to bacteremia (15). The indirect pathway depends on the significant source of inflammation determined by periodontitis, which, through the release of proinflammatory mediators such as IL-6, C-reactive protein and TNF alpha, leads to systemic inflammation (16, 17). Specifically, regarding atherosclerosis, persistent microbial infections in the vessel walls create a pro-inflammatory environment, potentially triggering autoimmune responses against vascular cells and endothelial dysfunction, thereby initiating atherosclerosis (18, 19). Moreover, the intestinal microbiota has been shown to influence ischemic stroke episodes, and since the oral microbiota impacts the intestinal microbiota altering homeostasis and autoimmune defense mechanisms, this interaction, though not yet fully elucidated, could influence ischemic events (20). The 2020 consensus report by the European Federation of Periodontology (EFP) and the World Heart Federation (WHF), representing an update from the 2012 workshop by the EFP and the American Academy of Periodontology, confirms that there is evidence from epidemiological studies for a positive association between periodontitis and cerebrovascular disease. The relative risk estimates vary between studies, depending on population characteristics and periodontitis case definitions (14, 21). However, some studies have found no significant relationship between periodontal disease and cerebrovascular stroke (22, 23). Zheng et al. (19) reported that only severe and moderate periodontitis was associated with an increased risk of stroke, whereas for mild periodontitis the association was not statistically significant. There remains a lack of comprehensive studies based on large populations and precise gradations of periodontitis examining the relationship between periodontitis and stroke (24). Therefore, the aim of this systematic review and meta-analysis, was to ascertain and quantify the actual impact of periodontal disease in individuals with cerebrovascular stroke, through the analysis of periodontal inflammatory indices. This study seeks to elucidate the potential mechanisms linking periodontal disease to stroke and to provide a comprehensive assessment of the current evidence in this field.

## 2 Materials and methods

### 2.1 Protocol and registration

The systematic review was composed following the PRISMA guidelines (Preferred Reporting Items for Systematic Reviews and Meta-Analyses) (25). All research procedures, selection, and data extraction adhered to the directives outlined in the Cochrane Handbook, and the review protocol was submitted and registered on the PROSPERO platform with registration number CRD42024529767.

### 2.2 Eligibility criteria

All potentially eligible case-control studies pertaining to cerebral strokes that re-ported data specifically on periodontal inflammatory indices and, more broadly, on periodontal health, were considered. The formulated PICO question was as follows: What is the impact of periodontal disease in patients with cerebral stroke compared to non-affected patients; (P) participants (patients with cerebral stroke); (I) intervention (presence of periodontal disease), (C) control (patients without cerebral stroke); and (O) outcome (differences in the impact of periodontal disease in patients with cerebral stroke compared to controls).

The inclusion criteria were: case-control studies reporting data on periodontal inflammation indices in patients with cerebral stroke. The exclusion criteria were as follows: excluding all reports related to systematic reviews, narrative reviews, case reports, *in vitro* and in silico studies, studies that did not report data on periodontal disease, studies published in a language other than English, and those at high risk of bias.

### 2.3 Sources of information, research and selection

The studies were identified through bibliographic searches on electronic databases by two authors (M.D. and A.B.). Restrictions on the language of publication were applied, and articles in languages other than English were excluded. Bibliographic searches were conducted on databases PubMed, Scopus, and Cochrane library. The last literature search was conducted on April 1st, 2024. Additionally, a search of Grey literature was also conducted by consulting Google Scholar, Science Direct, and Open Gray, and the bibliographic sources of previous systematic reviews on the topic were also investigated.

For the database search, the following terms in combination were adopted: stroke, periodontitis, and tooth. The following search terms were searched on PubMed:

Search: Stroke AND (periodontitis OR Tooth); Sort by: Most Recent;

(“stroke”[MeSH Terms] OR “stroke”[All Fields] OR “strokes”[All Fields] OR “strokes”[All Fields]) AND (“periodontal”[All Fields] OR “periodontally”[All Fields] OR “periodontically”[All Fields] OR “periodontics”[MeSH Terms] OR “periodontics”[All Fields] OR “periodontic”[All Fields] OR “periodontitis”[MeSH Terms] OR “periodontitis”[All Fields] OR “periodontitides”[All Fields] OR (“teeths”[All Fields] OR “teeths”[All Fields] OR “tooth”[MeSH Terms] OR “tooth”[All Fields] OR “teeth”[All Fields] OR “tooth s”[All Fields] OR “tooths”[All Fields]));

Translations: Stroke: “stroke”[MeSH Terms] OR “stroke”[All Fields] OR “strokes”[All Fields] OR “stroke's”[All Fields];

Periodontitis: “periodontal”[All Fields] OR “periodontally”All Fields] OR “periodontically"[All Fields] OR “periodontics”[MeSH Terms] OR “periodontics”[All Fields] OR “periodontic”[All Fields] OR “periodontitis”[MeSH Terms] OR “periodontitis”[All Fields] OR “periodontitis”[All Fields];

Tooth: “teeth's”[All Fields] OR “teeths”[All Fields] OR “tooth”[MeSH Terms] OR “tooth”[All Fields] OR “teeth”[All Fields] OR “tooth's”[All Fields] OR “tooths”[All Fields].

On the Scopus and Cochrane Library platforms, the following search terms and criteria were used: TITLE-ABS-KEY: stroke AND (periodontitis OR tooth).

Duplicates were removed using EndNoteX8 and manually (duplicate records not eliminated by the software were manually removed after the screening phase of the studies). The identified articles were independently evaluated and reviewed by two reviewers (M.D. and A.B.). The assessment of potentially eligible articles was based on the title and abstract, while the full text was evaluated for inclusion in the systematic review. Additionally, the agreement (kappa) between the two reviewers was assessed, and any disagreements were resolved by a third reviewer (S.C).

### 2.4 Data collection process and data characteristics

The type of data and information to be extracted was previously determined by the two authors responsible for article screening and transcribed independently into tables for subsequent comparison, aiming to minimize and reduce the risk of bias.

The extracted data from the articles included the first author, year of publication, study type, country conducting the study, number of patients, mean age, gender, Gingival index (GI), Plaque index (PI), Bleeding on probing [*n*, (%)] (BOP), Radiological bone loss (%, mm) (RBL), Probing pocket depth (mm) (PPD), Clinical attachment loss (mm) (CAL), and the presence of risk factors and comorbidities (hypertension, smoking, diabetes mellitus, hyperlipidemia, previous stroke-transient ischemic attack, alcohol drinking, coronary heart disease, peripheral arterial disease, atrial fibrillation).

### 2.5 Risk of bias within individual studies, summary measures, summary of results, risk of bias between studies, publication bias and additional measures

The Newcastle-Ottawa Quality Assessment Scale for case-control studies was used to measure the risk of bias and was evaluated by the two authors (A.B. and M.D.) responsible for selecting the studies. Studies with a high risk of bias were excluded from the systematic review and meta-analysis.

The results were extracted and reported in tables, while aggregated data were represented in figures such as forest plots with respective numerical values of Mean Difference (MD) and indices of heterogeneity like the Higgins index (*I*^2^).

Bias between studies was visually assessed (funnel plot and forest plot) by analyzing the overlaps of confidence intervals (C.I.), through the inconsistency index *I*^2^ (a value of *I*^2^ greater than 30% was considered moderate and a random-effects analysis was applied in specific cases), as well as through a funnel plot. If the meta-analysis showed high indices of heterogeneity, a sensitivity analysis was performed by excluding only studies with low C.I. overlap or those visually evident from the funnel plot.

For the meta-analysis, specifically for MD, Review Manager 5.4 software (Cochrane Collaboration, Copenhagen, Denmark) was used. The online software GRADE pro-Guideline Development Tool (GRADE pro-GDT, Evidence Prime) and TSA (Trial Sequential Analysis) utilizing Java-based software, TSA software (Copenhagen Trial Unit, Center for Clinical Intervention Research, Copenhagen, Denmark) were also conducted.

## 3 Results

### 3.1 Selection of studies

The research question guiding the study selection was as follows: What is the impact of periodontal disease in patients with cerebral stroke compared to non-affected patients?

The search phase was conducted by consulting and extracting bibliographic references from two databases, SCOPUS (2,040 records) and PubMed (1,398 records), and from the Cochrane Library registry (189 studies), resulting in a total of 3,627 records. The references were uploaded to EndNote X8, and duplicates were removed using software, resulting in 2,381 records. Any duplicates not identified by the software were manually detected and removed after the screening phase.

**Figure 1:**
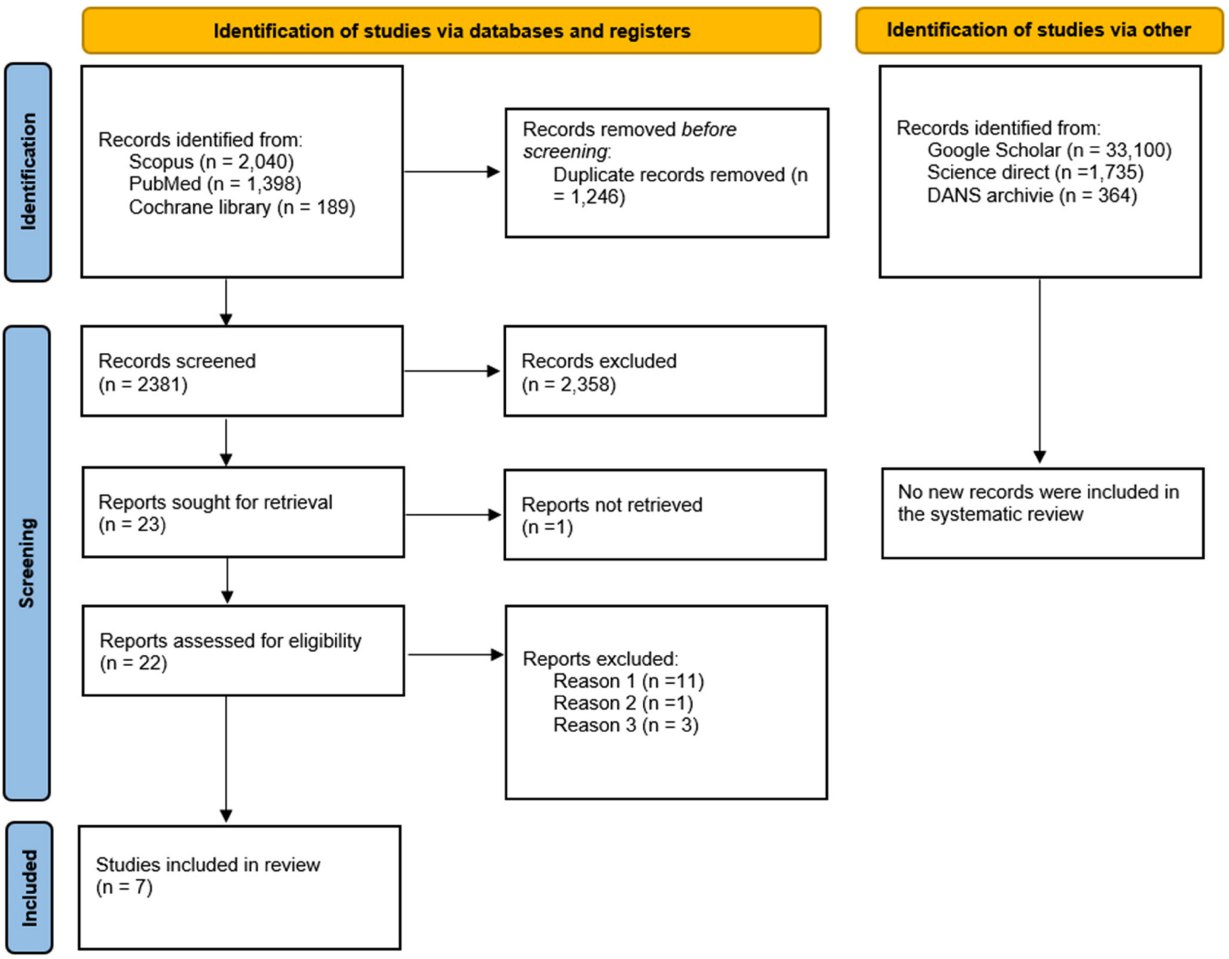
Flowchart of the article selection process; DANS, data archiving and networked services) reason 1: studies which, despite having a control cohort, are conducted prospectively, reason 2: case control studies reporting data on a population that has already been investigated and included: excluded to avoid overlapping data, reason 3: studies excluded because they assessed the presence of periodontal disease without reporting data on the indicators. Ogura et al. 2011 [7], Sim et al. [8] and Palm et al. 2014 [9].

After reviewing the titles and abstracts of the records, an equal number of 23 potentially eligible articles were identified, and at the end of the selection process, a total of 9 articles were included for quality assessment (Figure 1).

Further grey literature searches conducted on Google Scholar, Science Direct, and OpenGray using the keywords “stroke AND periodontitis AND case control” and “Stroke” on DANS archive did not yield additional studies to include in the review (Figure 1). The records were screened by two authors (M.D. and A.B.) independently, and any ambiguous situations were addressed at the end of the selection process by involving a third author (S.C.) to resolve potential conflicts.

### 3.2 Data characteristics

The articles included in the review are 7 and are as follows: Dörfer et al. (26), Pradeep et al. (27), Abolfazli et al. (28), Lafon et al. (29), Diouf et al. (30), Ghizoni et al. (31) and Leira et al. (32).

The extracted data is presented in two tables. Table 1 represents data regarding the first author, the country of the study, the total number of patients in different groups, the mean age or range, gender, and the presence of risk factors and comorbidities (Hypertension, Smoking, Diabetes mellitus, Hyperlipidemia, Previous stroke-transient ischemic attack, Alcohol drinking, Coronary heart disease, Peripheral arterial disease, Atrial fibrillation).

**Table 1:** Data on the characteristics of the affected patients and on the coexistence of morbidità.

First author, data	Country	Group	Age mean, DS	Sex (F, M)	Hyp	Sm	Dm, HyLi, Ps	Al	CoHe, PeAr, AtF
Dörfer et al. (26)	Germany	303 case	59.7 ± 11.2	95, 208	172	98a Ne, 95 Ex-Sm, 110 Cu	71, 109, 86	110 no, 171 Mod, 22 Heavy	46, 34, 21
		300 control	59.3 ± 8.0	87, 213	102	150 Ne, 87 Ex-Sm, 63 Cu	21, 90, 10	90 no, 102 Mod, 8 Heavy	19, 8, 4
Pradeep et al. (27)	India	100 case	52.3 ± 8.1	44, 56	65	24 Ne, 20 Ex-Sm, 56 Cu	60, \, \	22 no, 25 O, 53 Re	\
		100 control	51.7 ± 9.2	44, 56	32	44 Ne, 21 Ex-Sm, 35 Cu	42, \, \	22 no, 40 O, 38 Re	\
Abolfazli et al. (28)	Iran	100 case ischemic stroke	54.2 ± 12.15	48, 52	61	17 Cu	17	\	\,9, 9
		50 hospital staff, control	52.6 ± 14.28	27, 23	15	10 Cu	12	\	\, 7, 4
		50 hospitalized non stroke, control	53.1 ± 12.61	27, 23	23	13 Cu	12	\	\, 6, 3
Lafon et al. (29)	France	48 case	60.2 ± 11.8	22, 26	24	17 Ne, 12 Cu, 19 Ex-Sm	9	18 no, 30 Re	6, 3, 10
		47 control	56.1 ± 8.8	26, 21	8	18 Ne, 19 Cu, 10 Ex-Sm	2	27 no, 20 Re	1, 1, 0
Diouf et al. (30)	Senegal	120 case	\	\	76.7%	8.3% Cu, 27.5% Pa-Sm	18.3%	8.3% Re	\
		120 control	\	\	22.5%	7.5% Cu, 35% Pa-Sm	10.8%	3.3% Re	\
Ghizoni et al. (31)	Brazil	20 case	59 ± 13	\	\	7 Cu	\	\	\
		60 control	48 ± 10	\	\	17 Cu	\	\	\
Leira et al. (32)	Spain	62 case	68.0 (58.0 −71.2)	25, 47	\	28 Cu	15	15 Re	9, 0, \
		60 control	68.0 (58.0 −71.0)	21, 39	\	11 Cu	6	6 Re	2, 0, \

DS, deviation standard; F female; M, male; Hyp, hypertension; Sm, smoking; Ne, never; Cu, current; Ex-Sm, Ex-smoker; Pa-Sm, passive smoker; Dm, diabetes mellitus; HyLi, hyperlipidemia; Ps, previous stroke-transient ischemic attack; Al, alcohol drinking; Mod, moderate; CoHe, coronary heart disease; PeAr, peripheral arterial disease; AtF, atrial fibrillation; O, occasional; Re, regular.

Table 2 presents data concerning the first author and the main indicators of periodontal disease: Gingival index, Plaque index, Bleeding on probing [*n*, (%)], Radiological bone loss (%, mm), Probing pocket depth (mm), and Clinical attachment loss (mm).

**Table 2:** Data on inflammatory periodontal indices.

First author, data		Gingival index	Plaque index	Bleeding on probing [*n*, (%)]	Radiological bone loss (%, mm)	Probing pocket depth (mm)	Clinical attachment loss (mm)
Dörfer et al. (26)	Case	0.97 ± 0.35	1.68 ± 0.60	\	24.13 ± 2.85 (3.82 ± 1.97)	4.04 ± 0.97	4.30 ± 1.33
	Control	0.68 ± 0.37	1.55 ± 0.51	\	20.17 ± 8.47 (3.28 ± 1.53)	3.72 ± 0.81	3.87 ± 1.18
Pradeep et al. (27)	Case	1.23 ± 0.31	1.73 ± 0.45	\	\	4.5 ± 1.16	3.99 ± 1.21
	Control	1.07 ± 0.38	1.48 ± 0.5	\	\	3.65 ± 0.86	3.18 ± 0.94
Abolfazli et al. (28)	Case	\	\	\	\	\	2.98 ± 1.97
	Control	\	\	\	\	\	2.14 ± 1.98
	Control	\	\	\	\	\	2.48 ± 2.11
Lafon et al. (29)	Case	2.68 ± 0.94	2.63 ± 1.01	39 (88)	18.1 ± 9	\	\
	Control	2.17 ± 1.14	2.12 ± 1.27	31 (67)	13.7 ± 6	\	\
Diouf et al. (30)	Case	\	1.9 ± 0.404	0.5 ± 0.374	\	2.7 ± 0.673	2.0 ± 1.657
	Control	\	1.4 ± 0.371	0.3 ± 0.287	\	2.4 ± 0.510	1.0 ± 0.913
Ghizoni et al. (31)	Case	\	1.0 ± 0.0	1.1 ± 0.2	\	2.6 ± 2.4	5.1 ± 4.4
	Control	\	1.8 ± 0.4	1.7 ± 0.8	\	2.4 ± 1.5	3.2 ± 2.6
Leira et al. (32)	Case	\	62.4 ± 19.1	67 (52.5–78)	\	5.0 ± 1.6	6.2 ± 2.1
	Control	\	37.1 ± 14.4	33 (26.2–38)	\	3.3 ± 1.0	3.8 ± 1.2

The study design was necessarily homogeneous, considering only case-control studies. The total number of patients included with cerebral stroke was 723, while controls numbered 787. Smokers in the case groups totaled 230, compared to 177 in the control groups. The mean age of the groups, approximately calculated, was around 56 years. All studies reported CAL, except for Lafon et al., (29), while PPD (mm) was investigated in 5 studies and RBL (%, mm) in 2 studies.

### 3.3 Risk of bias

The risk of bias was assessed as acceptable for all studies included in the systematic review. The studies were evaluated using the following parameters, referring to the Newcastle-Ottawa Quality Assessment Scale for case-control studies: Selection: Is the case definition adequate? Representativeness of the cases, Selection of Controls, Definition of Controls; Comparability: Comparability of cases and controls based on the design or analysis; Exposure: Ascertainment of exposure, Same method of ascertainment for cases and controls, non-response rate (Table 3).

**Table 3:** Risk of bias, NOS.

First author, data	Selection				Comparability	Exposure		
	Is the case definition adequate?	Representativeness of the cases	Selection of Controls	Definition of Controls	Comparability of cases and controls on the basis of the design or analysis	Ascertainment of exposure	Same method of ascertainment for cases and controls	Non-Response rate
Dörfer et al. (26)	+	+	+	+	++	+	+	+
Pradeep et al. (27)	+	+	+	+	++	+	+	+
Abolfazli et al. (28)	+	+	+	+	+	+	+	+
Lafon et al. (29)	+	+	+	+	+	+	+	+
Diouf et al. (30)	+	+	+	+	+	+	+	+
Ghizoni et al. (31)	+	+	+	+	+	+	+	+
Leira et al. (32)	+	+	+	+	+	+	+	+

### 3.4 Meta-analysis

The meta-analysis of the data was conducted using the Review Manager 5.4 software (Cochrane Collaboration, Copenhagen, Denmark), which was also utilized for generating the images of the forest plot and funnel plot.

The data extracted from the studies were divided into 3 different meta-analyses concerning the data on CAL, PPD, and RBL.

However, the data regarding GI, PI, and BOP were heterogeneous. Due to variability in measurement units and scale measurement variations, it was decided not to apply the Standard MD and consequently not include these data in further meta-analyses.

#### 3.4.1 Clinical attachment loss (CAL)

**Figure 2:**
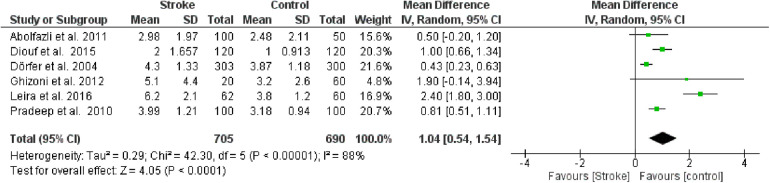
Forest plot of clinical attachment loss, mean difference: 1.04 95% CI [0.54, 1.54], Tau^2^ = 0.29, Higgins heterogeneity index *I*^2^ = 88, Chi^2^ = 42.30, df (degrees of freedom) 5, *P* value < 0.00001, test for overall effect: *Z* = 4.05 (*P* < 0.0001) weights: Abolfazli et al. 2011 15.6%, Diouf et al. 2015 20.3%, Dörfer et al. 2004 21.6%, Ghizoni et al. 2012 4.8%, Leira et al. 2016 2.40%, Pradeep et al. 2010 20.7%.the graph for each study included shows the first author, the date of publication, the number of patients with stroke and control, the average clinical attack loss in the two groups with the standard deviation (SD), the mean difference, the weight of the study on the meta-analysis. The final effect of the single study is expressed in a green square with the related confidence intervals (black line crossing the square) while the final effect of the meta-analysis is depicted by the black diamond whose width is given by the confidence intervals.

The first meta-analysis conducted focused on CAL. Random effects were applied, and the MD between the control group (without stroke) and the stroke group was calculated. The aggregated MD value was 1.04 [0.54, 1.54], indicating a reduction in CAL favoring the control group compared to the stroke group. In fact, the central diamond representing the effect size did not intercept the line of no effect (Figure 2). The studies included in this meta-analysis were as follows: Abolfazli et al., 2011 [12], Diouf et al. 2015 [14], Dörfer et al. 2004 [10], Ghizoni et al. 2012[15], Leira et al. 2016 [16], Pradeep et al. 2010 [11]. For Abolfazli et al., 2011, data from the control group referring to hospitalized non-stroke patients were included in the meta-analysis. Only 2 studies intercept the line of no effect: Ghizoni et al. 2012 [15] and Abolfazli et al., 2011 [12].

**Figure 3:**
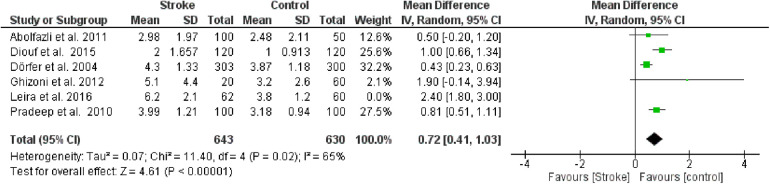
Sensitivity analysis, clinical attachment loss forest plot of the random effects model of the meta-analysis, exclusion of Leira et al., 2016 data.

Furthermore, a sensitivity analysis was conducted to identify the source of heterogeneity in the meta-analysis (Figure 3). Selectively excluding studies revealed that the source was the study by Leira et al. 2016 [16]. Indeed, excluding the data from this study reduced the Higgins *I*^2^ index from 88% to 65%.

#### 3.4.2 Probing pocket depth (PPD)

**Figure 4:**
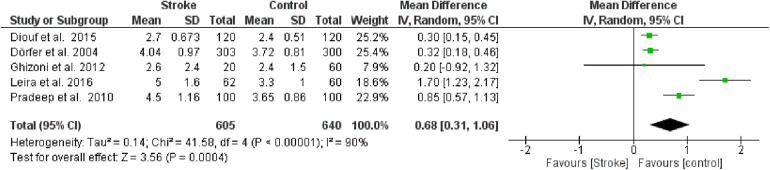
Forest plot of probing pocket depth, mean difference: 0.68 95% CI [0.31, 1.06], Tau^2^ = 0.14, Higgins heterogeneity index *I*^2^ = 90, Chi^2^ = 41.58, df (degrees of freedom) 4, *P* value <0.00001, test for overall effect: *Z* = 3.56 (*P* < 0.0004) weights: Diouf et al., 2015 25.2%, Dörfer et al., 2004 25.4%, Ghizoni et al., 2012 7.9%, Leira et al., 2016 18.6%, Pradeep et al., 2010 22.9%. The graph for each study included shows the first author, the date of publication, the number of patients with stroke and control, the average clinical attack loss in the two groups with the standard deviation (SD), the mean difference, the weight of the study on the meta-analysis. The final effect of the single study is expressed in a green square with the related confidence intervals (black line crossing the square) while the final effect of the meta-analysis is depicted by the black diamond whose width is given by the confidence intervals.

The second meta-analysis conducted utilized data on PPD. Random effects were also applied in this analysis, and the MD between the control group (without stroke) and the stroke group was calculated. The aggregated MD value was 0.68 [0.31, 1.06], indicating a reduction in PPD favoring the control group compared to the stroke group. In fact, the central diamond representing the effect size did not intercept the line of no effect. The studies included in this meta-analysis were as follows: Diouf et al. (30), Dörfer et al. (26), Ghizoni et al. (31), Leira et al. (32), Pradeep et al. (27). Only the study by Ghizoni et al. (31) intercepted the line of no effect (Figure 4).

**Figure 5:**
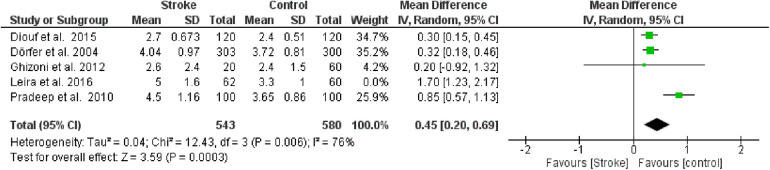
Sensitivity analysis, probing pocket depth forest plot of the random effects model of the meta-analysis, exclusion of Leira et al., 2016 data.

Furthermore, a sensitivity analysis was conducted to identify the source of heterogeneity in the meta-analysis. Selectively excluding studies revealed that the source was the study by Leira et al. 2). Indeed, excluding the data from this study reduced the Higgins *I*^2^ index from 80% to 76% (Figure 5).

#### 3.4.3 Radiological bone loss (RBL)

**Figure 6:**
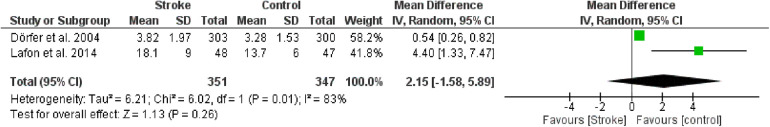
Forest plot of radiological bone loss, mean difference: 2.15 95% CI [−1.58, 5.89], Tau^2^ = 6.21, Higgins heterogeneity index *I*^2^ = 83, Chi^2^ = 6.02, df (degrees of freedom) 1, *P* value = 0.01, test for overall effect: *Z* = 1.13 (*P* = 0.26) weights: Dörfer et al., 2004 58.2%, Lafon et al., 2014 41.8%, the graph for each study included shows the first author, the date of publication, the number of patients with stroke and control, the average clinical attack loss in the two groups with the standard deviation (SD), the mean difference, the weight of the study on the meta-analysis. The final effect of the single study is expressed in a green square with the related confidence intervals (black line crossing the square) while the final effect of the meta-analysis is depicted by the black diamond whose width is given by the confidence intervals.

The third meta-analysis conducted pertained to Radiological Bone Loss. Fixed effects were applied in this analysis, and the MD between the control group (without stroke) and the stroke group was calculated. The aggregated MD value was 2.15[−1.58, 5.89] indicating a slight reduction in RBL favoring the control group compared to the stroke group. Indeed, the central diamond representing the effect size catch the line of no effect (Figure 6).

Only two studies were included in this meta-analysis: Dörfer et al. (26) e Lafon et al. (29). Given the low number of included studies, it was decided not to perform any further analysis.

### 3.5 Publication bias

**Figure 7:**
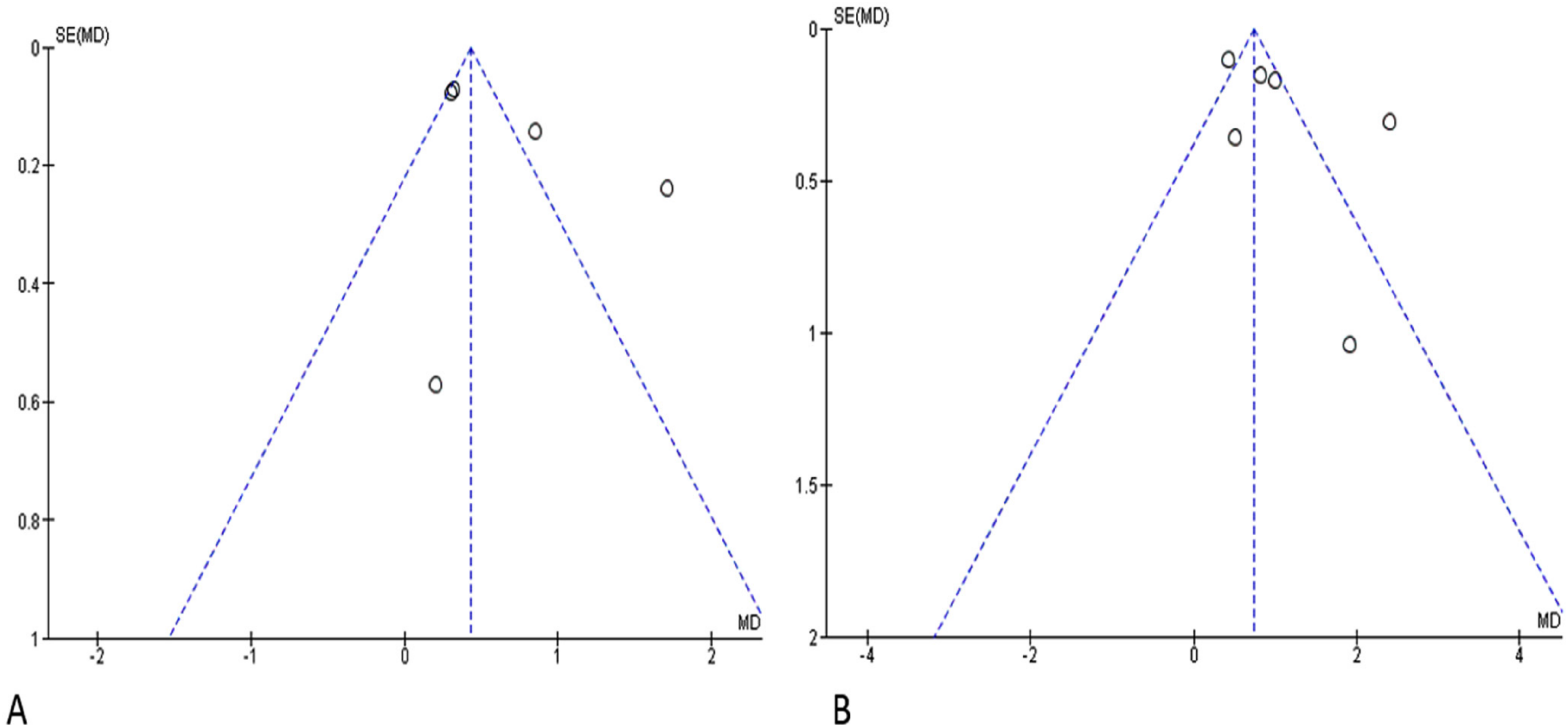
Funnel plot. SE, standard error; MD, mean difference; **(A)** clinical attachment loss; **(B)** probing pocket depth.

An assessment of Publication Bias was also conducted through visual analysis of the distribution of studies on the funnel plots, where an asymmetry in the data distribution was observed for both graphs (Figure 7A: Funnel plot for Clinical Attachment Loss, Figure 7B: Funnel plot for Probing Pocket Depth).

### 3.6 Trial sequential analysis

**Figure 8:**
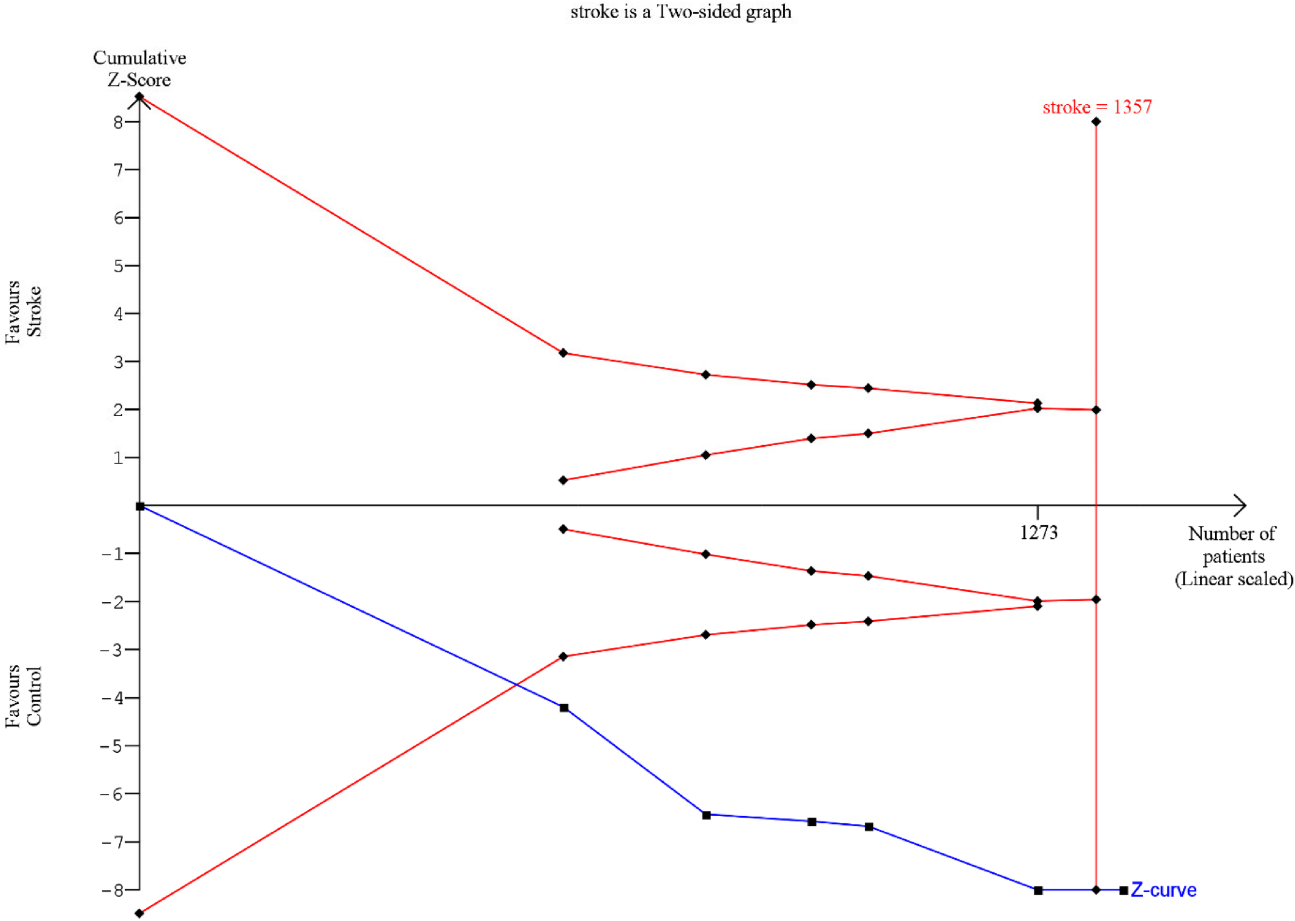
TSA: red lines represent the sequential trial monitoring limits and futility limits. The solid blue line is the cumulative *Z*-curve that requires the information dimension to demonstrate or reject a 20% relative reduction in clinical attachment loss in stroke patients compared to controls (5% alpha and 80% beta). After the first study the cumulative *Z*-statistic crossed above 1.96, which corresponds to the nominal threshold for statistical significance, using conventional techniques.

A Trial sequential analysis (TSA) was executed o estimate the potency of the result of the first metanalysis, (Clinical attachment loss) and by adjusting the results to avoid type II and I errors. The program used was TSA free software. The O'Brien–Fleming spending function was utilized by applying fixed effects; for the purpose of determining the power of the results, an alpha value of 5% (type 1 error), and a beta value of 80% (type 2 error) were used (Figure 8).

### 3.7 Grade

The authors also used GRADE pro-GDT to assess the quality of the evidence on the meta-analysis. The results suggest that the quality of evidence is moderate (Table 4).

**Table 4:** Grade clinical attachment loss, probing pocket depth, radiological bone loss, ⊕◯◯◯ very low, ⊕⊕◯◯ Low, ⊕⊕⊕◯ moderate, ⊕⊕⊕⊕ high.

Certainty assessment							No of patients		Effect	Certainty
№ of studies	Study design	Risk of bias	Inconsistency	Indirectness	Imprecision	Other considerations	Stroke	Controls	(95%CI)	
Clinical attachment loss										
6	Case control study	Not serious	Not serious	Not serious	Not serious	All plausible residual confounding would reduce the demonstrated effect	705	609	MD 0.73 (0.59–0.88)-	⊕⊕⊕◯ Moderate
Probing pocket depth										
5	Observational studies	Not serious	Not serious	Not serious	Not serious	All plausible residual confounding would reduce the demonstrated effect	605-	640	MD 0.43 (0.33–0.52)	⊕⊕⊕◯ Moderate
Radiological bone loss										
2	Case control study	Not serious	Not serious	Not serious	Not serious	All plausible residual confounding would reduce the demonstrated effect	351	347	MD 0.57 (0.29–0.85)	⊕⊕⊕◯ Moderate

## 4 Discussion

The authors conducted a comprehensive systematic literature review aimed at identifying and quantifying the actual impact of periodontal disease on individuals who have experienced a stroke. This was achieved through an in-depth analysis of periodontal indices. Specifically, meta-analyses were performed on three of the primary periodontal disease indices: CAL, PPD, and RBL. Furthermore, Trial Sequential Analysis was utilized to evaluate the robustness and reliability of the meta-analysis results. The review encompassed seven case-control studies, incorporating data from a total of 723 stroke patients and 787 control subjects.

Both periodontal disease and stroke involve inflammatory processes in their etiopathogenesis. It has been hypothesized that the inflammatory response observed in patients with periodontitis may contribute to an increased risk of ischemic stroke (33). Bacteria involved in periodontal disease have the ability to enter the bloodstream and cause transient bacteremia. This condition may contribute to the onset and progression of atherosclerosis and ultimately cause transient ischemia or stroke (34). Specifically, the membranes of Gram-negative bacteria, such as *Aggregatibacter actinomycetemcomitans* and *Porphyromonas gingivalis*, contain lipopolysaccharides, which can trigger a systemic inflammatory response characterized by elevated levels of interleukins (IL-1beta, IL-6), C-reactive protein (CRP), and tumor necrosis factor-alpha (TNF-alpha) (35, 36). The released cytokines amplify inflammation by stimulating the proliferation of inflammatory cells within the arteries. However, numerous details regarding the pathophysiological mechanisms underlying this association remain largely unknown (37).

One of the primary confounding factors for periodontal disease evaluated in the included studies is smoking, which is a significant risk factor for both periodontal disease and stroke (19). Despite this, many studies have found that periodontitis remains independently associated with stroke even after adjusting for smoking (38, 39). Cerebral ischemia and periodontal disease share several common vascular risk factors, including hypertension, diabetes mellitus, hypercholesterolemia, and alcohol consumption (40).

The association between periodontitis and stroke has been previously suggested in the literature. For instance, Leira et al. (41), in a review of 8 studied, 5 case-control and 3 cohort, demonstrated a statistically significant association (relative risk of 2.88) between ischemic stroke and periodontitis,. Their study provided powerful evidence that periodontal disease could be a significant risk factor for stroke, highlighting the importance of oral health in the context of systemic diseases.

This finding was later corroborated by a recent systematic review and meta-analysis conducted by Fagundes et al. in (8), which separated 7 case-control studies from 3 cohort studies in their quantitative analysis, reporting a risk of 2.31 (95% CI: 1.39–3.84; *I*^2^ = 77%; *p* = 0.0003) and 1.88 (95% CI: 1.55–2.28; *I*^2^ = 0%; *p* = 0.37), respectively.

It is necessary to consider prospective and case-control studies separately because the literature analysis clearly indicates that case-control studies are suitable for evaluating potential short-term associations between diseases such as periodontitis and rapidly progressing conditions like stroke. However, these studies, compared to the prospective ones, are insufficient to establish a temporal relationship between exposure to periodontal disease and the onset of stroke. In these studies, the periodontal status was retrospectively assessed after the occurrence of a stroke, while control subjects were selected from populations within a specific age range, chosen with heightened awareness of their overall health status. This selection process introduces an inherent bias when assessing periodontal disease as a potential risk factor for stroke. This bias must be considered in our review, as only case-control studies were included (33).

The data from our meta-analysis reveal a worsening of periodontal indices in stroke patients compared to control subjects, in accordance with the evidence in the literature (8). The aggregated MD for the CAL was 1.04 mm (95% CI 0.54–1.54, *p*-value < 0.0001), while for PPD, it was 0.68 mm (95% CI 0.31–1.06, *p*-value < 0.00001). Although RBL was only assessed in two of the included studies, the MD was found to be 2.15 mm (95% CI −1.58 to –5.89, *p*-value < 0.001). Despite graphical evidence of publication bias, the data were statistically significant and demonstrated good power, as illustrated by the TSA. Furthermore, it must be taken into account that some cases could be potentially overestimated or underestimated due to the intrinsic measurement errors of periodontal clinical parameters such as CAL and PPD, which have a margin of error of ±1 mm (42, 43).

These findings conclusively demonstrate the impact of cerebrovascular stroke on periodontal health. The loss of clinical attachment, coupled with radiological bone loss, indicates not only an increase in inflammatory periodontal indices but also significant damage to the periodontium, which is closely related to the health conditions of stroke patients (44, 45).

Given these findings, early interventions aimed at maintaining oral and periodontal health in patients who have experienced a cerebrovascular stroke should be planned and integrated into their overall rehabilitation and support interventions. It has been demonstrated that maintaining oral hygiene in subjects with cerebrovascular stroke can reduce adverse events and improve their quality of life (46, 47). It is important to consider that the reduction in motor, sensory, and cognitive abilities in stroke patients may be one of the main causes of the decline in their oral hygiene (48). This bidirectional relationship between periodontal disease and systemic conditions highlights the importance of comprehensive management and prevention strategies that address both oral and general health (49). Therefore, patients and their caregivers should be educated and motivated to perform dental hygiene maneuvers, possibly with the assistance of chlorhexidine mouthwashes (50). They should also be made aware of the importance of understanding and evaluating potential risks associated with poor oral hygiene (48).

## 5 Conclusions

The systematic review and meta-analyses conducted in this study confirmed the significant impact of periodontal disease on stroke patients. In conclusion, this study provides valuable information on the relationship between periodontal disease and stroke, underscoring the importance of oral health in cerebrovascular health outcomes. Further research and targeted interventions are needed to fully exploit these findings to improve stroke prevention and management strategies. The findings therefore highlight the importance of early interventions to maintain oral and periodontal health in individuals with stroke. Given the incidence of periodontal disease in cerebrovascular stroke patients, integrating oral hygiene measures into post-stroke rehabilitation programs could potentially reduce the incidence of adverse events and improve the overall quality of life of stroke survivors.

## Data Availability

The original contributions presented in the study are included in the article/Supplementary Material, further inquiries can be directed to the corresponding author.
