# Corrigendum: Impact of cerebrovascular stroke on inflammatory periodontal indices: a systematic review with meta-analysis and trial sequential analysis of case-control studies

**DOI:** 10.3389/froh.2025.1580261

**Published:** 2025-03-13

**Authors:** Mario Dioguardi, Maria Eleonora Bizzoca, Stefania Cantore, Giorgia Apollonia Caloro, Gennaro Musella, Filiberto Mastrangelo, Lorenzo Lo Muzio, Andrea Ballini

**Affiliations:** ^1^ Department of Clinical and Experimental Medicine, University of Foggia, Foggia, Italy; ^2^ Department of Precision Medicine, University of Campania “Luigi Vanvitelli”, Naples, Italy; ^3^ Unità Operativa Nefrologia e Dialisi, Presidio Ospedaliero Scorrano, ASL (Azienda Sanitaria Locale) Lecce, Scorrano, Italy

**Keywords:** stroke, periodontitis, brain, oral and dental health, bone loss, oral inflammation, risk factor, tooth loss

A corrigendum on Impact of cerebrovascular stroke on inflammatory periodontal indices: a systematic review with meta-analysis and trial sequential analysis of case-control studies By Dioguardi M, Bizzoca ME, Cantore S, Caloro GA, Musella G, Mastrangelo F, Lo Muzio L, Ballini A. Front Oral Health. (2024) 5:1473744. doi: 10.3389/froh.2024.1473744

Error in Figure/Table

In the published article, there was an error in figures 2–6 as published. In the forest plot images (figures 2–6) representing the results of the meta-analysis that have been published, the fixed effects have been applied unlike the Random effects as described in the captions and in the manuscript. The corrected (Figures 2–6) and its caption.

**Figure 2:**
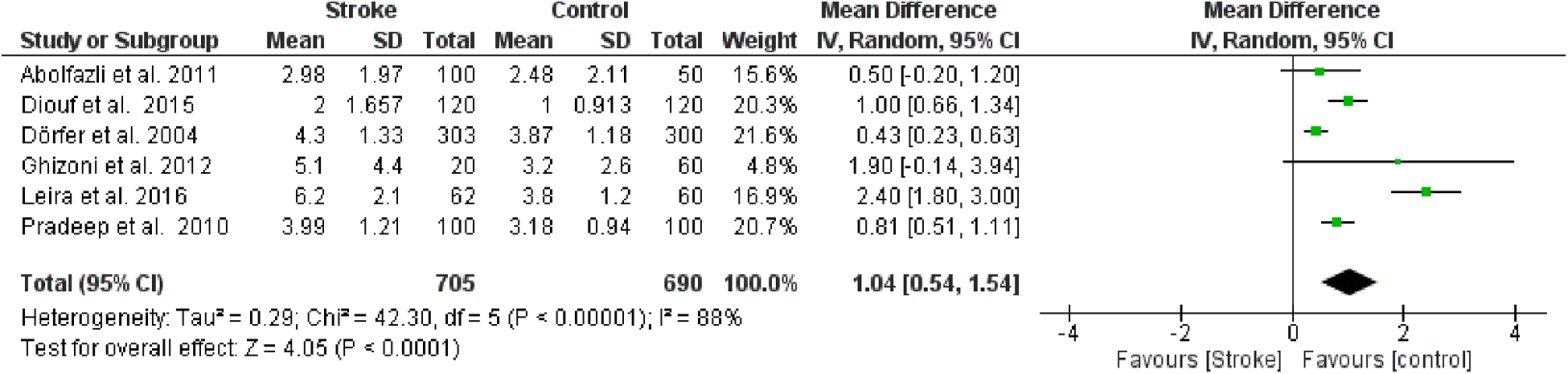
Forest plot of clinical attachment loss, mean difference: 1.04 95% CI [0.54, 1.54], Tau^2^ = 0.29, Higgins heterogeneity index *I*^2^ = 88, Chi^2^ = 42.30, df (degrees of freedom) 5, *P* value < 0.00001, test for overall effect: *Z* = 4.05 (*P* < 0.0001) weights: Abolfazli et al. 2011 15.6%, Diouf et al. 2015 20.3%, Dörfer et al. 2004 21.6%, Ghizoni et al. 2012 4.8%, Leira et al. 2016 2.40%, Pradeep et al. 2010 20.7%.the graph for each study included shows the first author, the date of publication, the number of patients with stroke and control, the average clinical attack loss in the two groups with the standard deviation (SD), the mean difference, the weight of the study on the meta-analysis. The final effect of the single study is expressed in a green square with the related confidence intervals (black line crossing the square) while the final effect of the meta-analysis is depicted by the black diamond whose width is given by the confidence intervals.

**Figure 3:**
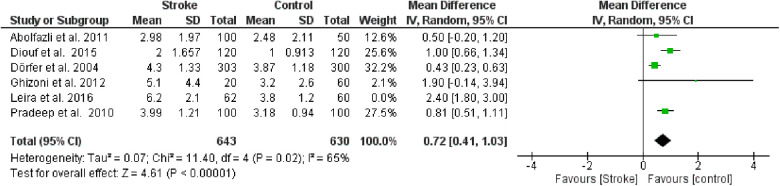
Sensitivity analysis, clinical attachment loss forest plot of the random effects model of the meta-analysis, exclusion of Leira et al., 2016 data.

**Figure 4:**
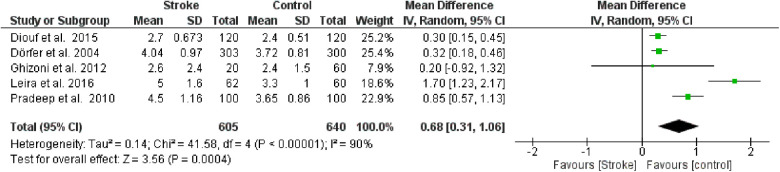
Forest plot of probing pocket depth, mean difference: 0.68 95% CI [0.31, 1.06], Tau^2^ = 0.14, Higgins heterogeneity index *I*^2^ = 90, Chi^2^ = 41.58, df (degrees of freedom) 4, *P* value <0.00001, test for overall effect: *Z* = 3.56 (*P* < 0.0004) weights: Diouf et al., 2015 25.2%, Dörfer et al., 2004 25.4%, Ghizoni et al., 2012 7.9%, Leira et al., 2016 18.6%, Pradeep et al., 2010 22.9%. The graph for each study included shows the first author, the date of publication, the number of patients with stroke and control, the average clinical attack loss in the two groups with the standard deviation (SD), the mean difference, the weight of the study on the meta-analysis. The final effect of the single study is expressed in a green square with the related confidence intervals (black line crossing the square) while the final effect of the meta-analysis is depicted by the black diamond whose width is given by the confidence intervals.

**Figure 5:**
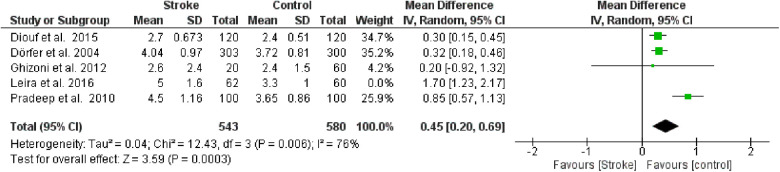
Sensitivity analysis, probing pocket depth forest plot of the random effects model of the meta-analysis, exclusion of Leira et al., 2016 data.

**Figure 6:**
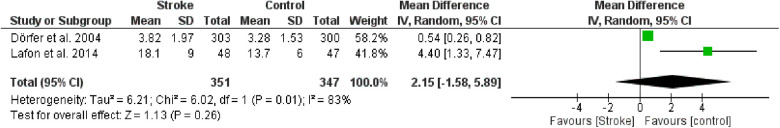
Forest plot of radiological bone loss, mean difference: 2.15 95% CI [−1.58, 5.89], Tau^2^ = 6.21, Higgins heterogeneity index *I*^2^ = 83, Chi^2^ = 6.02, df (degrees of freedom) 1, *P* value = 0.01, test for overall effect: *Z* = 1.13 (*P* = 0.26) weights: Dörfer et al., 2004 58.2%, Lafon et al., 2014 41.8%, the graph for each study included shows the first author, the date of publication, the number of patients with stroke and control, the average clinical attack loss in the two groups with the standard deviation (SD), the mean difference, the weight of the study on the meta-analysis. The final effect of the single study is expressed in a green square with the related confidence intervals (black line crossing the square) while the final effect of the meta-analysis is depicted by the black diamond whose width is given by the confidence intervals.

ORIGINAL Figures 2–6 appear below.

**Figure 2:**
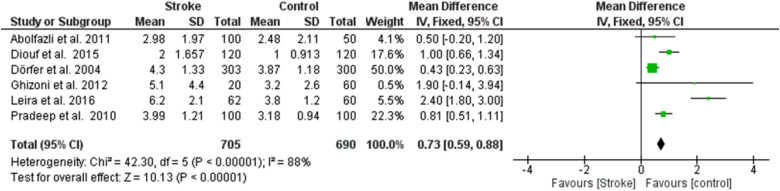
Forest plot of clinical attachment loss, mean difference: 1.04 95% CI [0.54, 1.54], Tau^2^ = 0.29, Higgins heterogeneity index *I*^2^ = 88, Chi^2^ = 42.30, df (degrees of freedom) 5, *P* value < 0.00001, test for overall effect: *Z* = 4.05 (*P* < 0.0001) weights: Abolfazli et al. 2011 15.6%, Diouf et al. 2015 20.3%, Dörfer et al. 2004 21.6%, Ghizoni et al. 2012 4.8%, Leira et al. 2016 2.40%, Pradeep et al. 2010 20.7%.the graph for each study included shows the first author, the date of publication, the number of patients with stroke and control, the average clinical attack loss in the two groups with the standard deviation (SD), the mean difference, the weight of the study on the meta-analysis. The final effect of the single study is expressed in a green square with the related confidence intervals (black line crossing the square) while the final effect of the meta-analysis is depicted by the black diamond whose width is given by the confidence intervals.

**Figure 3:**
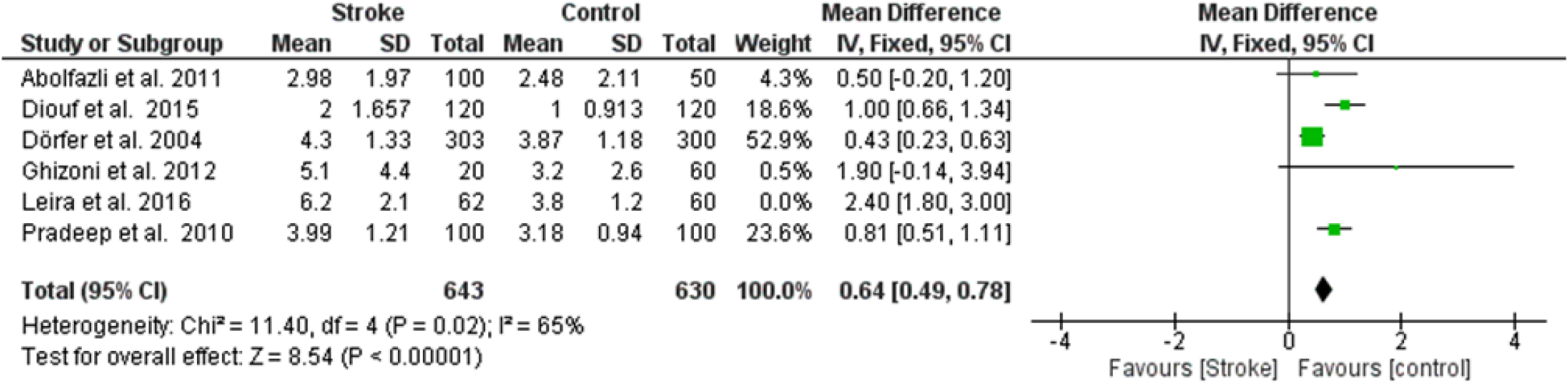
Sensitivity analysis, clinical attachment loss forest plot of the random effects model of the meta-analysis, exclusion of Leira et al., 2016 data.

**Figure 4:**
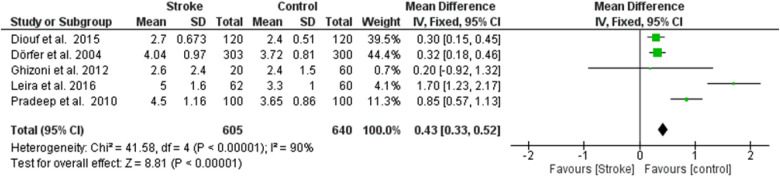
Forest plot of probing pocket depth, mean difference: 0.68 95% CI [0.31, 1.06], Tau^2^ = 0.14, Higgins heterogeneity index *I*^2^ = 90, Chi^2^ = 41.58, df (degrees of freedom) 4, *P* value <0.00001, test for overall effect: *Z* = 3.56 (*P* < 0.0004) weights: Diouf et al., 2015 25.2%, Dörfer et al., 2004 25.4%, Ghizoni et al., 2012 7.9%, Leira et al., 2016 18.6%, Pradeep et al., 2010 22.9%. The graph for each study included shows the first author, the date of publication, the number of patients with stroke and control, the average clinical attack loss in the two groups with the standard deviation (SD), the mean difference, the weight of the study on the meta-analysis. The final effect of the single study is expressed in a green square with the related confidence intervals (black line crossing the square) while the final effect of the meta-analysis is depicted by the black diamond whose width is given by the confidence intervals.

**Figure 5:**
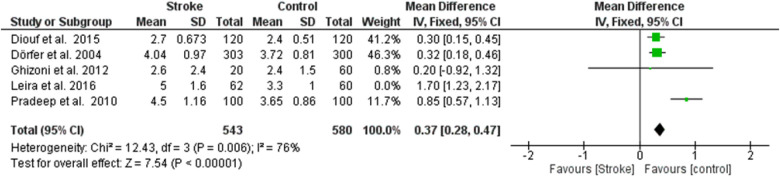
Sensitivity analysis, probing pocket depth forest plot of the random effects model of the meta-analysis, exclusion of Leira et al., 2016 data.

**Figure 6:**
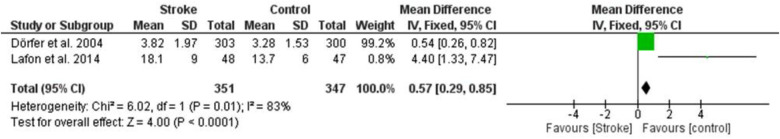
Forest plot of radiological bone loss, mean difference: 2.15 95% CI [−1.58, 5.89], Tau^2^ = 6.21, Higgins heterogeneity index *I*^2^ = 83, Chi^2^ = 6.02, df (degrees of freedom) 1, *P* value = 0.01, test for overall effect: *Z* = 1.13 (*P* = 0.26) weights: Dörfer et al., 2004 58.2%, Lafon et al., 2014 41.8%, the graph for each study included shows the first author, the date of publication, the number of patients with stroke and control, the average clinical attack loss in the two groups with the standard deviation (SD), the mean difference, the weight of the study on the meta-analysis. The final effect of the single study is expressed in a green square with the related confidence intervals (black line crossing the square) while the final effect of the meta-analysis is depicted by the black diamond whose width is given by the confidence intervals.

The authors apologize for this error and state that this does not change the scientific conclusions of the article in any way. The original article has been updated.

